# Supplementary material for: Development of an Online Mind–Body Physical Activity Intervention for Young Adults during COVID-19: A Pilot Study
Source: Int J Environ Res Public Health. 2023 Mar 4;20(5):4562. doi: 10.3390/ijerph20054562 (PMC10002143; doi:10.3390/ijerph20054562)
Supplement: Supplementary file 1 [file ijerph-20-04562-s001.zip › ijerph-2188564-supplementary.pdf]

Table S1. The chronological summary of the MBPA intervention procedures.

					**DATA COLLECTION**
**MODULE 1**	**WEEK 1**	1st<br>10-minute Session:<br><br>Breathing Activity: Deep Breathing	2nd<br>10-minute Session:<br><br>Breathing Activity: Deep Breathing	3rd<br>10-minute Session:<br><br>Breathing Activity: Ocean Breath	**Before starting week one** breathing activities, participants will complete the **Pre-Intervention Phase surveys at time-point 1, 2, and 3** (WHO-5, PSS-4, Interoception).<br><br>The pre-intervention-phase lasts for approximately 3 weeks.<br><br>Schedule to pick up your accelerometer and wear it for 7 days (baseline data).
	WEEK 2	4th<br>10-minute Session:<br>Breathing Activity: Ocean Breath	5th<br>10-minute Session:<br>Breathing Activity:<br>4-7-8	6th<br>10-minute Session:<br>Breathing Activity:<br>4-7-8	
**MODULE 2**	**WEEK 3**	7th<br>10-minute Session:<br>Ancient Movement Activities	8th<br>10-minute Session:<br>Ancient Movement Activities	9th<br>10-minute Session:<br>Ancient Movement Activities	**TIME-POINT 4**<br>Complete the 1st Intervention Phase surveys (WHO-5, PSS-4, Interoception).<br>Schedule to drop off your accelerometer at your earliest convenience.<br>Optional: 1-hour Zoom meeting
	WEEK 4	10th<br>10-minute Session:<br>Ancient Movement Activities	11th<br>10-minute Session:<br>Ancient Movement Activities	12th<br>10-minute Session:<br>Ancient Movement Activities	
**MODULE 3**	**WEEK 5**	13th<br>10-minute Session:<br>Walking Activities	14th<br>10-minute Session:<br>Walking Activities	15th<br>10-minute Session:<br>Walking Activities	**TIME-POINT 5**<br>Complete the 2nd Intervention Phase surveys (WHO-5,<br>PSS-4, Interoception).<br>Schedule to pick up your accelerometer and wear it for 7 days (midpoint data).<br>Optional: 1-hour Zoom meeting
	WEEK 6	16th<br>10-minute Session:<br>Walking Activities	17th<br>10-minute Session:<br>Walking Activities	18th<br>10-minute Session:<br>Walking Activities	Schedule to drop off your accelerometer at your earliest convenience.
**MODULE 4**	**WEEK 7**	19th<br>10-minute Session:<br>Loving-Kindness Activities	20th<br>10-minute Session:<br>Loving-Kindness Activities	21st<br>10-minute Session:<br>Loving-Kindness Activities	**TIME-POINT 6**<br>Complete the 3rd Intervention Phase surveys (WHO-5,<br>PSS-4, Interoception).<br><br>Optional: 1-hour Zoom meeting
	WEEK 8	22nd<br>10-minute Session:<br>Loving-Kindness Activities	23rd<br>10-minute Session:<br>Loving-Kindness Activities	24th<br>10-minute Session:<br>Loving-Kindness Activities	Schedule to pick up your **accelerometer and wear it for 7 days** (postintervention data).<br>Schedule to drop off your accelerometer at your earliest convenience.
